# Perception of surgical complications among patients, nurses and physicians: a prospective cross-sectional survey

**DOI:** 10.1186/1754-9493-5-30

**Published:** 2011-11-22

**Authors:** Ksenija Slankamenac, Rolf Graf, Milo A Puhan, Pierre-Alain Clavien

**Affiliations:** 1 Department of Surgery, University Hospital Zurich, Switzerland; 2 Horten Center for Patient Oriented Research, University Hospital Zurich, Switzerland; 3 Department of Epidemiology, Johns Hopkins Bloomberg School of Public Health, Baltimore, USA

**Keywords:** Perception, surgical complications, patients, nurses and physicians

## Abstract

**Background:**

Several scores grade the severity of post-operative complications but it is unclear whether such scores truly reflect the perception of patients and practicing nurses and physicians.

**Study Design:**

227 patients, 143 nurses and 245 physicians independently rated the severity of 30 common post-operative complications on a numerical analogue scale from 0 (not severe at all) to 100 (extremely severe) while being blinded towards the Clavien-Dindo classification. We considered a difference in ratings of >10 to be clinically important in distinguishing between grades of severity and groups. We evaluated the level of reproducibility of responses by calculating intraclass correlation coefficients (ICC) and compared scores across severity grades and between groups using the generalized estimating equations.

**Results:**

Reproducibility of the ratings was good for all three groups (ICCpatients 0.71 (95%-CI 0.64-0.76), ICCnurses 0.83 (0.78-0.87) and ICCphysicians 0.87 (0.83-0.90)). The participants' perceptions of the severity of complications reflected the Clavien-Dindo classification (median of grade I: 20 (IQR 10-30), grade II: 40 (31.3-52.5), grade IIIa: 50 (40-60), grade IIIb: 70 (60-75), grade IVa: 85 (80-90) and grade IVB: 95 (90-100)). Although patients' perception differed significantly from those of physicians (average difference -8.7 (95%-CI -10.4 to -6.9, p < 0.001) and nurses (difference -2.8 (-4.8 to -0.8, p = 0.007) they did not reach our thresholds for clinical importance.

**Conclusions:**

The severity of post-operative complications is perceived similarly by patients, nurses and physicians and reflects the Clavien-Dindo classification well. Our results support the use of Clavien-Dindo classification system as part of the shared or informed decision making process.

## Introduction

A well-known editorial in the Lancet highlighted the poor methodology and lack of convincing outcome measures in most surgical studies [1]. Mortality and a variety of markers for morbidity are commonly used but there is an ongoing debate on how to define and standardize post-operative complications. This is well illustrated by a systematic review that found more than 40 different definitions of anastomotic leaks in 107 different studies [2]. Additionally, terms such as major, severe or minor complications were used in an inconsistent manner, often without any explicit definition.

A group of surgeons, epidemiologists, and statisticians recognized these shortcomings and published a series of articles [3-5]. In 1992, Clavien et al proposed new definitions for post-operative complications, and a classification system to grade complications by severity based on the type and invasiveness of the treatment needed to treat a complication [6]. In 2004, a revised version of the classification system was proposed, based on the same principle, but eliminating criteria such as length of hospital stay [7]. This revised Clavien-Dindo classification classifies post-operative complications from grade I to V according to their need for more or less invasive treatment [7].

However, all attempts to classify postoperative complications were developed by experts without taking the perspective of patients and practising health care professionals into consideration. If the perception of the severity of postoperative complication is weakly associated with a classification system its use in research and practice seems to be of limited value. In turn, if a classification system reflects the perspective of patients and health care professionals there would be opportunities to use the classification system for research but also to explain potential risks for post-operative morbidity to patients and, thereby, to support the decision making process. In the absence of evidence in the literature we assessed how patients, nurses and physicians perceive the severity of post-operative complications and how strongly their perception is associated with the Clavien-Dindo classification system for post-operative complications.

## Materials and methods

### Study design, Population

**Figure 1:**
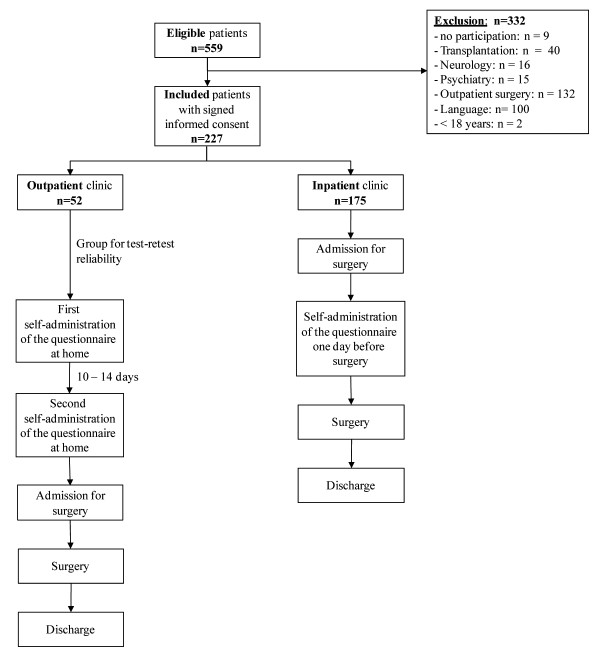
**Study flow**.

We conducted a prospectively planned cross-sectional study and included physicians, nurses and patients between January 21 and December 20, 2009. We invited patients scheduled for elective minor or major abdominal surgery with any underlying disease at a single tertiary care centre (Department of Visceral and Transplantation Surgery, University Hospital of Zurich, Switzerland). Further inclusion criteria were capacity to act without legal guardian and spoken and written German as the daily language. Patients were excluded if they had cognitive difficulties and diseases, which may result in unreliable answers, and if they were unable to read and/or write. We recruited eligible patients giving informed consent from the inpatient clinic completing the questionnaires once before surgery a well as from the outpatient clinic completing the questionnaire twice in order to assess its reproducibility (Figure 1).

We recruited nurses and physicians from surgical departments (visceral surgery, general surgery, traumatology and urology) and disciplines closely related to surgery (anesthesia and surgical intensive care units). We also sent the questionnaire to heads of surgical departments of 63 hospitals in the German-speaking part of Switzerland, 29 hospitals in Germany and 4 hospitals in Austria and invited them to participate voluntarily and anonymously with their surgical teams in this survey. Since it was not possible to retrieve the exact number of nurses and surgeons in those teams we could not calculate the response rate in nurses and physicians.

The study was approved by the institutional review board of Zurich (Switzerland) for human studies and internationally registered at clinicaltrials.gov (NCT 00785096).

### Development of the questionnaire

We first identified a total of 161 post-operative complications in abdominal surgery from our prospectively managed database and the literature [8-10]. Five board certified senior staff surgeons checked the list for completeness and estimated independently the *frequency *as well as the *relevance *of these complications on a Likert-type scale from 1 (very infrequent respectively not relevant) to 6 (very frequent respectively very relevant). In order to select the five most frequent and most relevant complications within each complication grade of the Clavien-Dindo classification (grades I, II, IIIa, IIIB, IVa and IVb, see below) we multiplied the frequency by the relevance for each complication and selected those five complications with the highest product within each complication grade (I to IVB). This approach resulted in 30 post-operative complication scenarios representing the most relevant and most frequent complications. Some complications appeared repeatedly due to different therapeutic consequences (e.g. re-operation due to anastomotic insufficiency [grade IIIb] and multi-organ failure due to anastomotic insufficiency [grade IVb]). Each of the 30 clinical scenarios described the complications themselves, what patients may feel when having these complications, the adequate therapy to treat the complications and its consequences on the length of hospital stay and future health outcomes. The questionnaire did not comment on the health state after (partial) recovery or on the course of recuperation after the hospital stay. The questionnaire was not disease-or and surgery-specific and, therefore, applicable to any patient planned for abdominal surgery. The content of the questionnaire was identical for patients, physicians and nurses but adapted to appropriate terminology so that the scenarios were easily understandable for all three groups. The questionnaire required about 30 minutes to complete. The full questionnaire is available from the authors on request.

We also developed a numerical answer scale from 0 (not severe at all) to 100 (extremely severe) to estimate the severity of post-operative complications. Numerical answer scales with numbered intervals and anchors are known to yield the most reliable answers and to require the shortest time for completion as compared with other scales (e.g. visual analogue scale) [11-21].

In cognitive debriefings, fifteen volunteers (five from each group) completed the draft questionnaire and were interviewed for completeness and comprehensibility of the 30 scenarios. Based on this feedback we made minor changes in wording and completed the final version of the questionnaire.

### The Clavien-Dindo classification of post-operative complications

The Clavien-Dindo classification [7] is a widely used therapy-oriented classification system and classifies post-operative complications from grade I to V according to their need for treatment. A grade I complication is any deviation from the normal postoperative course without the need of further treatment. A grade II complication requires a pharmacological treatment. A grade III complication requires surgical intervention under local (grade IIIa) or under general anesthesia (grade IIIb). A patient with a grade IV complication has a life-threatening complication and requires ICU management. Grade V means the death of a patient. This Clavien-Dindo classification has currently been used in more than 350 studies from various surgical fields [22-28] and was also proposed as a possible gold standard to assess post-operative complications [4].

### Sample size

There are no standard rules to estimate required sample sizes for the validation of patient-, nurses and physicians-reported outcomes. To estimate sample size requirements for reproducibility (test-retest reliability) we expected an intraclass correlation coefficient (ICC) of 0.7 to indicate sufficient test retest reliability. To estimate the ICCs with good precision (95% confidence interval width of ± 0.1), we calculated we needed to include data from 52 patients, 52 nurses and 52 physicians [29]. This group size would be large enough to detect a difference of 5 points on the 0 to 100 rating scale, which we considered to be of potential clinical relevance to distinguish between grades and groups of participants. Assuming a standard deviation of 12.5, based on a pilot study [23], we needed 219 participants in each group (patient, nurses and physicians) to have a power of 80% at a significance level of 0.05 while expecting a drop out rate of 15%.

### Statistical Analyses

Our database did not have any missing values since we paid considerable attention to a complete collection of data. In a first step, we expressed the distribution of ratings using means and standard deviation or medians and interquartile ranges.

We calculated ICCs to assess the reproducibility of the ratings. We considered ICCs of 0.7 or higher to be sufficient to proceed with the main analysis. In the main analysis, we used generalized estimating equations (GEE) to compare whether ratings differed between severity grades (I to IVb) and between patients, nurses and physicians. We used GEE to take the clustered structure of our data into consideration, as every respondent rated each of the 30 scenarios. A statistical model not considering the clustered structure would lead to an underestimation of standard errors. Because of our large sample size small differences could be statistically significant but of little clinical relevance. Therefore, we also determined, before conducting the analyses, a difference of 5 points or lower in average rating to be clinically non-important in distinguishing between grades of severity and groups. We considered a difference between 5 and 10 to be of potential importance, one that would need further investigation, and a difference of more than 10 to be clinically important. We conducted all analyses using STATA (version 10, Stata Corp., College Station, Texas).

## Results

### Participants

During the study period, 559 patients were assessed for eligibility. Three hundred and thirty-two patients were not eligible and 227 patients gave the full consent for the study (Figure 1). One hundred and seventy-five patients completed the questionnaire, of which 52 patients completed the questionnaire twice before surgery for testing reproducibility (Figure 1). Two hundred patients had minor or major abdominal surgery whereas 27 patients (11.9%) suffered from a disease which did not require surgery or could not be operated due to different reasons such as age, the presence of risk factors (e.g. major cardiac disease) or some patients denied further surgical treatment.

Patient characteristics and further intra-operative results are summarized in Table 1. Almost a third of the patients (73 of 227 patients) already had at least one post-operative complication in their medical history. Post-operative outcomes will be reported in detail elsewhere.

**Table 1:** Patients' characteristics, intra-operative parameters and post-operative outcome

Patients' characteristics	Patients (n = 227)
Age (years)	54 (41-66)

Gender, male/female (%)	116/111 (51.1%/48.9%)

ASA score	2 (2-3)
- ≤ 2	149 (65.6%)
- >2	78 (34.4%)

Nutrition risk score	1 (0-2)
- <3	189 (83.3%)
- ≥3	38 (16.7%)

Benign/malign disease (%)	153/74 (67.4%/32.6%)

Pre-operative chemotherapy (%)	31 (13.7%)

Pre-operative radiotherapy (%)	19 (8.4%)

Body mass index (kg/m^2^)	25.5 (22.1-31.6)

	

**Intra-operative parameters**	**Patients (n = 200)**

Minor/major surgery (%)	133/67 (66.5%/33.5%)

Surgery time (minutes)	120 (83.8-200)

Blood transfusion (%)	4 (2%)

Blood loss (mL)	20 (5-100)

	

**Post-operative outcome**	**Patients (n = 200)**

Length of hospital stay (days)	7 (4-9)

Intensive care unit stay (%)	33 (16.3%)

Intensive care unit stay (days)	0 (0-1)

Mortality (%)	0%

Morbidity (%)	107 (53.5%)

All results in median and interquartile range (IQR)

ASA = American Society of Anesthesiologists

Despite four written reminders and requests for voluntary and anonymous participation, only 143 nurses participated in the study (Table 2). Fifty-two of the 143 nurses completed the questionnaire twice for evaluating reproducibility. The time period between the first and second survey varied between five days (minimum) and eight weeks (maximum). In addition, 245 physicians (Table 2) participated in the study, of which 52 completed the questionnaire twice.

**Table 2:** Nurses' and physicians' characteristics

	Nurses (n = 143)
Gender, male/female (%)	29/114 (20.3%/79.7%)

Years on the job	12 (1-41)

Specialization	
- abdominal surgery	57 (39.8%)
- intensive care unit	27 (18.9%)
- emergency	24 (16.8%)
- cardiac/vascular	18 (12.6%)
- others	17 (11.7%)

	

	**Physicians (n = 245)**

Gender, male/female (%)	167/78 (68.2%/31.8%)

Years on the job	9 (4-18)

Country	
- Switzerland	192 (78.4%)
- Germany	38 (15.5%)
- Austria	15 (6.1%)

Position	
- resident	114 (46.5%)
- chief resident	8 (3.3%)
- junior attending surgeon	68 (27.8%)
- senior attending surgeon	27 (11.0%)
- chief of service	28 (11.4%)

Specialization	
- general surgery	94 (38.4%)
- abdominal surgery	79 (32.2%)
- anesthesia	23 (9.4%)
- cardiac/vascular	10 (4.1%)
- others	39 (15.9%)

All results in median and interquartile range (IQR)

Others in nurses = anesthesia, traumatology, thoracic surgery and operating-room nurses

Others in physicians = internal medicine, urology, gynecology, thoracic surgery, traumatology, gastroenterology, orthopedic and pediatric surgery

### Reproducibility of the questionnaire

Reproducibility of the ratings was good for all three groups: ICCpatients 0.71 (95% CI: 0.64 to 0.76), ICCnurses 0.83 (95% CI: 0.78 to 0.87 and ICCphysicians 0.87 (95% CI: 0.83 to 0.90)).

### Perception of the severity of post-operative complications

The median severity rating of the 30 complication scenarios differed, from 10 (IQR 5-17.5) to 95 (IQR 90-100). Median ratings from scenarios in grade I varied from 10 to 30 (IQR 5-40), in grade II ratings from 35 to 50 (IQR 25-60) and in grade IIIa from 40 to 60 (IQR 30-70). With an increase in severity of the complications (≥ grade IIIb) we observed less variation in median and IQR but still an increase in severity from grade to grade according to the Clavien-Dindo classification (Table 3).

**Table 3:** Perception of the severity of post-operative complications

Complication	Treatment	Grade	Median	Interquartile range
Hypopotassemia	oral substitution of potassium	I	10	5-17.5

Oedema	diuretics	I	15	10-25

Dystelectasis	breath-physiotherapy	I	20	10-30

Postoperative nausea and vomiting	antiemetics	I	20	10-30

Wound infection	wound opened at the bedside, secondary wound healing	I	30	20-40

Local infection	antibiotics	II	35	25-50

Arrhythmia	medical treatment (e.g. beta-blockers)	II	40	30-55

Subileus	gastric tube, procinetics, antiemetics	II	45	30-60

Gastroparesis	gastric tube, procinetics, antiemetics	II	45	30-60

Upper GI-bleeding due to ulcer	medical treatment (e.g. PPI), blood substitution	II	50	35-60

Wound infection	wound closure in local anesthesia	IIIa	40	30-50

Pneumothorax	thoracic drain in local anesthesia	IIIa	50	40-60

Upper GI-bleeding due to ulcer	gastroscopy with local treatment of the ulcer bleeding, medical treatment (e.g. PPI), blood substitution	IIIa	50	40-65

Intra-abdominal abscess	drainage	IIIa	57.5	40-70

Deep venous thrombosis	lyses	IIIa	60	50-70

Wound infection	wound closure in full anesthesia	IIIb	40	30-55

Post-operative acute bleeding	blood substitution, surgical revision	IIIb	70	60-80

Infected bilioma	surgical revision	IIIb	70	60-80

Anastomotic insufficiency	surgical revision, re-anastomoses	IIIb	70	60-80

Mechanical ileus	surgical remove of adhesions	IIIb	70	55-80

Delirium	medicaments, intubation	IVa	80	70-90

Lung emboli	anticoagulation, intubation	IVa	80	70-90

Acute liver failure	medical substitution, ICU support	IVa	85	75-90

Acute renal failure	hemofiltration, ICU support	IVa	85	75-90

Anastomotic insufficiency	antibiotics, surgical revision, hemodynamic stabilization on the ICU	IVa	90	80-97.5

Low output syndrome	hemodynamic stabilization, hemofiltration	IVb	90	80-100

Post-operative acute bleeding	blood substitution, surgical revision, hemodynamic stabilization, hemofiltration	IVb	90	80-100

Colon ischemia	antibiotics, colon resection, hemodynamic stabilization, hemofiltration	IVb	95	90-100

ARDS	intubation, hemodynamic stabilization	IVb	95	85.6-100

Anastomotic insufficiency	antibiotics surgical revision, hemodynamic stabilization, hemofiltration	IVb	95	90-100

Local infection was defined e.g. as central venous infection; PPI = proton-pump-inhibition treatment; ARDS = Acute respiratory distress syndrome

### Association of the patients' and health care professional perception with the Clavien-Dindo classification

**Figure 2:**
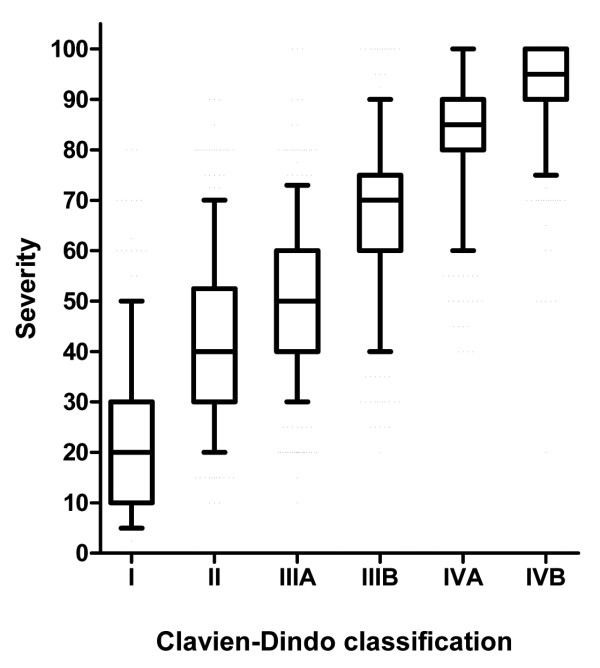
**Perception of the severity of post-operative complications from all participants: The questionnaire included 30 scenarios with five scenarios for each severity grade according to the Clavien-Dindo classification**. The box plots represent the median, interquartile range and 95%-confidence interval for all scenarios rated within each grade of the Clavien-Dindo classification. The perception of the severity of post-operative complications of all participants (patients, nurses and physicians) increases with the rise in the Clavien-Dindo classification.

**Figure 3:**
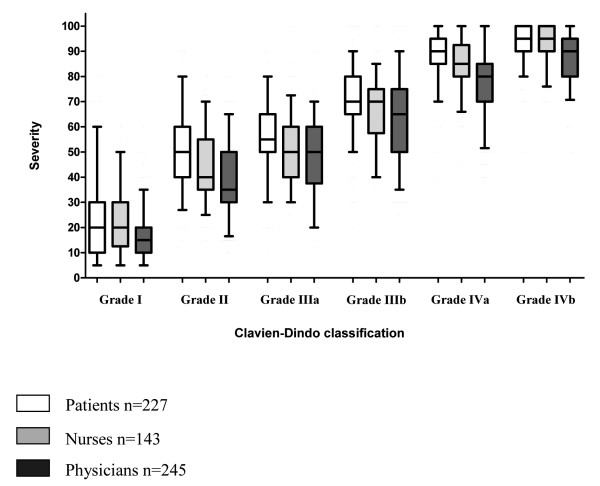
**Patients', nurses' and physicians' perception of the severity of post-operative complications: The box plots represented the median, interquartile range and 95%-confidence interval for all scenarios rated within each grade of the Clavien-Dindo classification and compares the three groups of participants (patients, nurses and physicians)**. Patients, nurses and physicians graded the complication scenarios similarly across all grades of the Clavien-Dindo classification (I to IVB) (Figure 3).

The perception of the severity of post-operative complications of all participants (patients, nurses and physicians) increased with the severity in the Clavien-Dindo classification (Figure 2). The ratings of all scenarios except for two (wound infections according to the grade IIIa and IIIb) matched with what would be expected from the Clavien-Dindo classification. Participants estimated the severity of a grade I complication with a median of 20 (IQR 10-30), grade II with a median of 40 (IQR 31.3-52.5), grade IIIA with a median of 50 (IQR 40-60), grade IIIB with a median of 70 (IQR 60-75), grade IVA with a median of 85 (IQR 80-90) and grade IVB complications with a median of 95 (IQR 90-100). Patients, nurses and physicians graded the complication scenarios similarly across all grades of the Clavien-Dindo classification (I to IVb) (Figure 3).

### Participants' perception of post-operative complications

Despite statistical significance, patients estimated the severity of post-operative complications similar to physicians with a difference that was below our a priori defined threshold for clinical relevance of >10 (unadjusted difference -8.7, 95%-confidence interval (CI) -10.4 to -6.9, p < 0.001) and nurses (unadjusted difference -2.8, 95%-CI -4.8 to -0.8, p = 0.007). Also, there was a statistically significant difference between the estimation of the severity between physicians and nurses (difference -5.9, 95%-CI -7.9 to -3.9, p < 0.001), which, however, did not exceed the threshold for clinical relevance.

## Discussion

Our study showed that the perception of the severity of post-operative complications can be measured reliably and that it closely reflects the Clavien-Dindo classification. Although the patients' perception differed statistically significantly from those of physicians and nurses we do not consider these differences to be clinically relevant.

For the first time a large number of patients, nurses and physicians estimated the perceived severity of the same complications. We compared these perceptions with a widely used classification system that was developed, based on the experience and knowledge of expert surgeons [2,7]. Although systems such as the Clavien-Dindo classifications system seemed to reduce uncertainty about how to define post-operative complications, the validity of this classification system was never evaluated from the perspective of patients and health care professionals. Notably, not only health care professionals but also patients classified the severity of complications according to the need for treatment and identically to the Clavien-Dindo classification. This is illustrated by the scenarios that refer to identical complications (e.g. anastomotic insufficiency, wound infection or ulcer) but different therapeutic consequences. We could also show that nurses, who often have a closer relationship with patients than physicians, perceive the severity of complications between that of physicians and patients, a result that is not unexpected.

The similarity in the perception of post-operative complications by patients and health care professionals indicate that they share a common basis, which could be valuable for decision making. Today, surgeons mainly explain the frequency of post-operative complications to patients without explaining their severity. Frequencies of possible post-operative complications are important but patients will be better informed if they also know their severity. Our study indicates that the Clavien-Dindo classification system could be used as the basis for an improved explanation to the patient, allowing the patient to better understand complications and their therapeutic consequences. This may greatly support the patients' decision for the proposed surgical treatment or alternatives. Since nurses share a similar perception about post-operative complications they may also get involved in the decision making process as equally important partners within the medical team.

### Strengths and limitations

The large sample size of 615 participants and the three different groups (patients, nurses and physicians) strengthened the results of this study. Moreover, we had no missing values, neither in the answers of the questionnaire nor in other ratings. However, we were not able to reach the planned sample size of 219 nurses. In order to avoid any circularity, this is that respondents were aware of the Clavien-Dindo classification for each vignette and would, therefore had made matching ratings, patients, physicians and nurses were blinded and also unaware of the Clavien-Dindo classification. Only twenty-five surgeons (out of 245) knew the Clavien-Dindo classification because they were from our surgical department but they were also blinded for our classification of each vignette.

Our study had also some potential limitations. Each complication scenario described a single complication and its consequences but we did not take into account that quite commonly more than one complication may occur. Further studies will need to assess how patients and health care professionals perceive multiple complications. Although we included a broad sample of participants, a possible selection bias could be that all our participants were from a university hospital. Participants from peripheral hospitals may grade the severity of post-operative complications differently. Further studies will also be needed on this topic.

## Conclusion

In conclusion, the perception of post-operative complications estimated by patients, nurses and physicians was similar and associated with the Clavien-Dindo complication classification. Our results lend support to the use of this classification system as part of the shared or informed decision making process.

## Abbreviations

ICC: intraclass correlation coefficient; GEE; generalized estimating equation; 95% CI; 95% confidence interval; IQR: interquartile range; ASA: American Society of Anesthesiologists; PPI: proton-pump-inhibition treatment; ARDS: acute respiratory distress syndrome.

## Competing interests

The authors declare that they have no competing interests.

## Authors' contributions

KS has made substantial contributions to conception and design, acquisition of data, analysis and interpretation of data. Furthermore KS has been involved in drafting the manuscript, revising it critically for important intellectual content and has given final approval of the version to be published.

RG has made substantial contributions to conception and design and interpretation of data: RG has been involved in revising it critically for important intellectual content and has given final approval of the version to be published.

MAP has made substantial contributions to conception and design, analysis and interpretation of data. Furthermore MAP has been involved in drafting the manuscript and revising it critically for important intellectual content. MAP has given final approval of the version to be published.

PAC has made substantial contributions to conception and design and interpretation of data. PAC has been involved in revising it critically for important intellectual content and has given final approval of the version to be published.
